# The Border Health Strategic Initiative From a Community Perspective^1^

**Published:** 2004-12-15

**Authors:** Victoria E Steinfelt

**Affiliations:** University of Arizona, Cooperative Extension

## A Community Coordinator's Perspective

As local community coordinator of the Border Health Strategic Initiative (*Border Health ¡SI!*), a program in Yuma County to develop a comprehensive, community-based approach to diabetes prevention and control, I was responsible for facilitating communication among five community partners, between community partners and campus-based faculty, and developing and coordinating a Special Action Group (SAG). My 30 years of experience as a cooperative extension agent, 27 of those years in Yuma County, helped to prepare me for this role.

As the Yuma County Cooperative Extension Agent, I had collaborated previously with each of the five community-based partners of *Border Health ¡SI!* on past programs, and I also had experience working with some of the campus-based faculty. *Border Health ¡SI!* provided funding to continue these partnerships and to build new partnerships with new campus faculty and community members. Twenty-five percent of my time was assigned to *Border Health ¡SI!,* and funding provided a full-time program assistant and half-time secretary for the cooperative extension component of the program. In addition to community coordination and the SAG, the Cooperative Extension Office was responsible for implementing the Centers for Disease Control and Prevention's School Health Index (SHI) in Yuma County (1).

The Cooperative Extension Office is located in the community, not on the university campus, and it involves local residents in identifying, planning, and implementing local programs. The Cooperative Extension Agent is a University of Arizona faculty member, thus providing a link between local community needs and university faculty expertise. This article addresses the details of coordinating a comprehensive diabetes prevention and management program from the viewpoint of a local community coordinator.

## Purpose and Setting of *Border Health ¡SI!*

*Border Health ¡SI! *was implemented in Arizona along the border between the United States and Mexico. In Yuma County, Arizona, the initiative served the municipalities of Somerton, Gadsden, and San Luis, representing a total population of 25,563 — 92.1% of which is Hispanic. Approximately 35% of the residents in these agricultural communities have incomes below the 200% poverty level, and 30% are without medical insurance (2). Additional information about the purpose and setting of *Border Health ¡SI!* can be found in the introductory article of this issue of *Preventing Chronic Disease* (3).

A team of 10 faculty members from the Mel and Enid Zuckerman Arizona College of Public Health worked with five community-based partners to plan, implement, and evaluate *Border Health ¡SI!.* The community partners were the Yuma County University of Arizona Cooperative Extension, Western Arizona Health Education Center, *Campesinos Sin Fronteras*, Sunset Community Health Center, and *Puentes de Amistad*. The intervention components, outcomes, and conclusions are described in the other articles in this issue of *Preventing Chronic Disease* (1,3-9).

## Recruiting Community Partners

**Timetable for Yuma County, Arizona, Special Action Group (SAG), The Border Health Strategic Initiative**

- November 2000 through January 2001: Recruitment of SAG members
- January 2001: First SAG meeting
- January to April 2001: Monthly meetings to identify issues
- April to June 2001: Subcommittees developed action plans
- June 2001: SAG approved action plans
- June 2002 to September 2003: Action plans implemented through *Border Health ¡SI!*

My first task as the community coordinator for the Border Health initiative was to organize a Special Action Group (1,9). Its mission was to determine and prioritize community policy issues surrounding physical activity and healthy eating. In November 2000, our small team (the Cooperative Extension program assistant and I) started the process of developing the SAG by making face-to-face contacts with key community leaders to explain the purpose and goal of *Border Health ¡SI!* and recruit them as members.

We did not set a goal for number of members to recruit because we were more interested in achieving broad community representation than meeting a numeric goal. For example, we recruited parks and recreation directors, city managers, community planners, school personnel, business owners, police officers, health care providers, county health department employees, elected officials, community residents, and others. The community partners were members of the SAG as well, and two campus-based faculty members participated in meetings and provided technical assistance to the SAG.

The face-to-face contacts were time consuming, but this strategy provided the best opportunity for a two-way dialogue about *Border Health ¡SI!* and the function of the SAG. During these dialogues, we asked for names of other key community people to contact. We also realized that community members lacked knowledge about diabetes prevention and discovered, ironically, that about half of those contacted had a family member with diabetes. We used this valuable information to prepare agenda items for the first SAG meeting, which took place in January 2001.

Once our team was established, our next major step was to schedule our first meeting, which took place two months after recruitment began. We met monthly for four months to select policy issues (9). Meeting notices were mailed each month to the 36 SAG members, and meeting attendance ranged from 20 to 28 members. During our fourth meeting, we formed two subcommittees to develop action plans. Each subcommittee had 10 to 12 members. One subcommittee developed the action plan to increase physical activity through advocating for more parks and walking paths, and the second committee addressed promoting healthier food choices in grocery stores and in schools (1). The entire group continued to meet monthly, and three months later we had action plans in place.

## The Challenge of Systematic Problem-solving

In my experience, when you bring together community members to address a problem or concern, the group wants to do something immediately. It is often a challenge to get community members to take the time to follow a systematic problem-solving process (10). This process includes 1) examining in depth the issues at hand; 2) identifying alternative ways to address the issues; 3) writing an action plan, including ways to evaluate and implement the plan; 4) evaluating progress; 5) modifying the plan if needed; and 6) assessing results.

Fortunately, we were able to follow the systematic problem-solving process with the SAG for several reasons. By the time the SAG first met, some *Border Health ¡SI!* interventions were already being implemented. Recruitment for the walking clubs and family component had started, and patient education classes were being taught, so our members had a feeling that something was happening in their community. Our university partners presented information about the incidence and burden of diabetes at our meetings and led a discussion about the difference between community interventions and policy issues. Once our members grasped the need to address policy, the group understood the benefit of taking time to plan.

One of the action steps in our plan was to address food selection by South Yuma County grocery stores. Low-fat and nonfat milk, diet soda, and yogurt, for example, were available in limited quantities or not available at all in some stores. We planned to provide healthy food-promotion booths in stores, featuring food choices highlighted in *Border Health ¡SI!* community nutrition classes. Our hope was that if more customers requested healthier food choices, the stores would stock these items. The idea of providing nutrition information at grocery stores broke new ground in South Yuma County. It took several months to schedule an appointment with the storeowner because of his busy schedule. The storeowner was somewhat reluctant to allow our program to provide samples and nutrition information in his stores. He did not want us to tell his customers not to buy certain foods. He asked for a written plan and list of foods the program would promote. Again, it took several months to schedule another appointment to present the plan and list of foods. After reviewing the plan, the owner allowed us to provide samples and nutrition information in his stores with the conditions that we purchased the supplies in his stores and that we would not tell customers not to buy certain foods. This process took approximately eight months.

## Surprises

Political changes in one community provided surprises. One of our action steps was to attend city council meetings in this community to support the open space and parks segment of the city plan, which was under discussion (9). A group of SAG members, walking club participants, and *promotoras* gathered to attend a city council meeting in which the city plan was listed as the first agenda item. When the group of 15 people arrived for the meeting, last-minute agenda issues arose because of a local political controversy, and the city plan was moved to the end of the meeting. Our group waited patiently for several hours but eventually went home before the city council introduced the item. The city plan item was scheduled for a later city council meeting, but we were able to gather only five people to attend.

When we implemented the SHI, we were surprised to discover the differences in beliefs among school principals regarding the role of schools in health promotion for students and staff. Some principals believed that the school has a very important role, and some believed that the school has no role. Obviously, it was easier to implement the SHI in the schools where the principal was supportive than in the schools where the principal was allowing us to implement the SHI but was not supportive. Through the SHI process, an action plan is developed, and the principal's support and encouragement for changes in the school is a critical element for changes to occur.

We were also surprised to learn about differences among community agencies regarding operating procedures, workplace culture, and funding sources. For example, operating procedures at the University of Arizona are different from those of a community-based nonprofit agency. The university's hiring procedures and expenditure process are more complicated, so it took longer for the university to hire program personnel and to obtain approval for program expenditures.

The differences in workplace culture revolved around the methods by which nonprofit agencies assigned personnel to *Border Health ¡SI!*. Some agencies cross-trained personnel who were funded by several different grants, while other agencies assigned total program responsibility to one person. Both strategies were effective, but at first it was confusing to know who exactly was working on *Border Health ¡SI!*.

We also discovered that the university could not serve as the lead organization for some grant applications. Some funds were available only to community-based nonprofit agencies. Small communities like ours tend to lack experienced proposal writers among nonprofit agencies, city offices, schools, and Cooperative Extension offices. The partnership between the local communities and the University of Arizona — newly strengthened by *Border Health ¡SI!* — provided community agencies with access to the university's proposal-writing expertise. Nonprofit agencies were able to apply for funding with technical assistance from the campus-based partner. Additionally, some nonprofit agencies and schools contracted with professional proposal writers, and the local community foundation offered proposal-writing training to community agencies.

## Results-yielding Synergy

Community groups begin simply as a collection of people. It takes time to evolve into a working team that generates synergy (11). Over the three years of the *Border Health ¡SI!*, our SAG members developed into a working team that had an impact on the community in a way that no one agency or organization could have accomplished working alone.

For example, in one small community, a community leader had been working for many years to renovate an existing park. He formed a community group and experienced some success. This individual was identified as a potential member of the parks and open space subcommittee of our SAG, and he agreed to join. At his first meeting, he described the frustrations of trying to obtain funding for the park and mentioned that he was just about ready to give up. The group listened to his concerns and empathized with his frustration. Members pointed out the accomplishments of his group and made a commitment to work together with this community leader and other community groups to increase the number of parks and renovate existing parks in South Yuma County. About a year later, after our first *Border Health ¡SI!* Park Development Community Forum, which was a component of the SAG's action plan, this person shared with me his appreciation for being a member of the SAG. He said that he and his community group would not have been able to reach their goals without the assistance of other SAG members.

During one meeting, our university partners led the SAG in a discussion to identify the outcomes the members felt they had accomplished. One comment made during the meeting summarizes the synergy that developed: "The SAG has been instrumental in bringing key government people to meetings and networking. Education of the government entities from this awareness and the funding for two parks has been acquired. Yuma County officials would not be open to listen or cooperate as much if the SAG hadn't been involved on a big scale."

## Conclusion

The Border Health Strategic Initiative was a three-year program that ended September 2003. The basic model that was developed will be continued and expanded through Steps to a HealthierUS, which is an initiative of the U.S. Department of Health and Human Services.

Although coordinating a comprehensive community-based health promotion program is time consuming, the synergistic relationship that evolves will yield exciting and rewarding results for you and the communities involved.
